# Rear‐edge, low‐diversity, and haplotypic uniformity in cold‐adapted *Bupleurum euphorbioides* interglacial refugia populations

**DOI:** 10.1002/ece3.6700

**Published:** 2020-08-24

**Authors:** Won‐Bum Cho, Soonku So, Eun‐Kyeong Han, Hyeon‐Ho Myeong, Jong‐Soo Park, Seung‐Hyun Hwang, Joo‐Hwan Kim, Jung‐Hyun Lee

**Affiliations:** ^1^ Department of Biology Education Chonnam National University Gwangju Korea; ^2^ Ecosystem Research Division Korea National Park Research Institute Wonju Korea; ^3^ Department of Biological Sciences and Biotechnology Chonnam National University Gwangju Korea; ^4^ Department of Biological Sciences Inha University Incheon Korea; ^5^ Department of Biology Daejeon University Daejeon Korea; ^6^ Department of Life Science Gachon University Seongnam‐si Korea

**Keywords:** Baekdudaegan, *Bupleurum euphorbioides*, cold‐adapted, genetic diversity, interglacial refugia, rear‐edge

## Abstract

The high genetic diversity of rear‐edge refugia populations is predicted to have resulted from species repeatedly migrating to low latitudes during glacial periods over the course of Quaternary climate change. However, several recent empirical studies of cold‐tolerant plants revealed the opposite pattern. We investigated whether current habitats of the cold‐adapted and range‐restricted *Bupleurum euphorbioides* in the Baekdudaegan, South Korea, and North Korea could be interglacial refugia, and documented how their rear‐edge populations differ genetically from those of typical temperate species. Phylogeographic analysis and ecological niche modeling (ENM) were used. The genetic structure was analyzed using microsatellite markers and chloroplast DNA sequences. The congener *B. longiradiatum* was included as a typical temperate plant species. Despite having almost identical life history traits, these congeneric species exhibited contrasting patterns of genetic diversity. ENM revealed an apparent maximum range contraction during the last interglacial. In contrast, its range expanded northward to the Russian Far East (Primorsky) during the Last Glacial Maximum. Thus, we hypothesize that *B. euphorbioides* retreated to its current refugia during interglacial periods. Unlike populations in the central region, the rear‐edge populations were genetically impoverished and uniform, both within populations and in pooled regional populations. The rear‐edge *B. euphorbioides* survived at least one past interglacial, contributing to the species’ genetic diversity. We believe that such genetic variation in the cold‐adapted *B. euphorbioides* gives the species the necessary adaptations to survive an upcoming favorable environment (the next glacial), unless there is artificial environmental change.

## Graphical abstract

The study shows a pattern that contrasts with the changes in the distribution range that typical temperate plants experienced in the course of Quaternary climate change. ENM revealed an apparent maximum range contraction during the last interglacial. In contrast, its range expanded northward to Russian Far East (Primorsky) during the Last Glacial Maximum. The rear‐edge populations were genetically impoverished and uniform, both within populations and in pooled regional populations. We believe that such genetic variation in the cold‐adapted *Bupleurum euphorbioides* gives it the necessary adaptations that will enable it to survive an upcoming favorable environment (the next glacial), unless there is artificial environmental change.

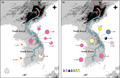

## 1 INTRODUCTION

Genetic structure in extant plant populations is known to be affected by various factors, including the diversity of pollinators, reproductive systems, seed dispersal modes, and historical migration patterns; historical range change during Quaternary climatic oscillations is considered a primary factor (Petit et al., 2003). Although their relative importance may vary across time and space, the genetic features of populations in northeastern Asian temperate regions likely reflect historical, rather than current, levels of gene flow (Aizawa, Kim, & Yoshimaru, 2012; Bao et al., 2015; Chen et al., 2012; Tamaki et al., 2019). Quaternary glacial periods have repeatedly forced temperate plant species to retreat southward, where they remained for long periods (Hewitt, 1996, 2000).

Rear‐edge populations are typically described as low‐latitude glacial refugia, providing long‐term stores of genetic diversity with high levels of variation and uniqueness (Provan & Maggs, 2011). Such genetic determinants, acquired via repeated adaptation processes, may play a key role in guaranteeing long‐term survival (Gugger, González‐Rodríguez, Rodríguez‐Correa, Sugita, & Cavender‐Bares, 2011; Lepais et al., 2013). Therefore, understanding rear‐edge population genetic diversity is likely to become a conservation research priority; it will provide a framework for assessing the ability of natural populations to adapt to changing environmental conditions (Provan & Maggs, 2011; Scalfi, Piotti, Rossi, & Piovani, 2009).

Many studies on glacial refugia have considered the genetic diversity of temperate, usually warm‐adapted, species (Petit, Hu, & Dick, 2008). This may be because there are relatively few cold‐adapted temperate plant species. Furthermore, the term “glacial refugia” may be too broad, as it does not adequately convey the wide range of behavioral patterns. Quaternary refugia are regions that species inhabit during the maximum range contraction in the glacial/interglacial cycle (Stewart, Lister, Barnes, & Dalén, 2010). Several empirical studies in East Asia have explained reasonably well how this occurs, accounting for the time frame of the maximum range contraction during the Quaternary (e.g., for *Rosa sericea*: Gao, Zhang, Gao, & Zhu, 2015; *Chrysanthemum indicum*: Li, Wan, Guo, Abbott, & Rao, 2014; and *Pinus kwangtungensis*: Tian, López‐Pujol, Wang, Ge, & Zhang, 2010). Nonetheless, our understanding of the genetic history and structure of rear‐edge populations occurring in cryptic southern refugia remains to be elucidated.

Baekdudaegan, a main mountain range of the Korean Peninsula, is a key ecological axis of northeastern Asia; it is connected to the Sikhote‐Alin mountains in the Russian Far East and the Lesser Khingan Range in China, spanning ca. 1,600 km from South Korea. It is one of the longest mountain chains in Asia, with 14 subsidiary mountain ranges; it is home to many boreal and temperate species, as well as to widespread and range‐restricted species (Chung, López‐Pujol, & Chung, 2016). The range is also a well‐known hotspot for biodiversity, particularly for threatened species (Chung et al., 2018). Although most of it remains relatively pristine, some parts of Baekdudaegan in South Korea are threatened. Its high conservation value has made it the site of many genetic studies, which have provided valuable information for conserving threatened species (Chang, Kim, & Park, 2003; Chang, Kim, Park, & Maunder, 2004; Jeong et al., 2010). Some studies have focused on the history of population establishment (Chung, Chung, López‐Pujol, Park, & Chung, 2013; Chung et al., 2012). As an important refugium, the parts of Baekdudaegan in South Korea are thought to have provided a stable habitat for the boreal and temperate species of northeastern Asia during glacial periods (Chung, López‐Pujol, & Chung, 2017).

**FIGURE 1:**
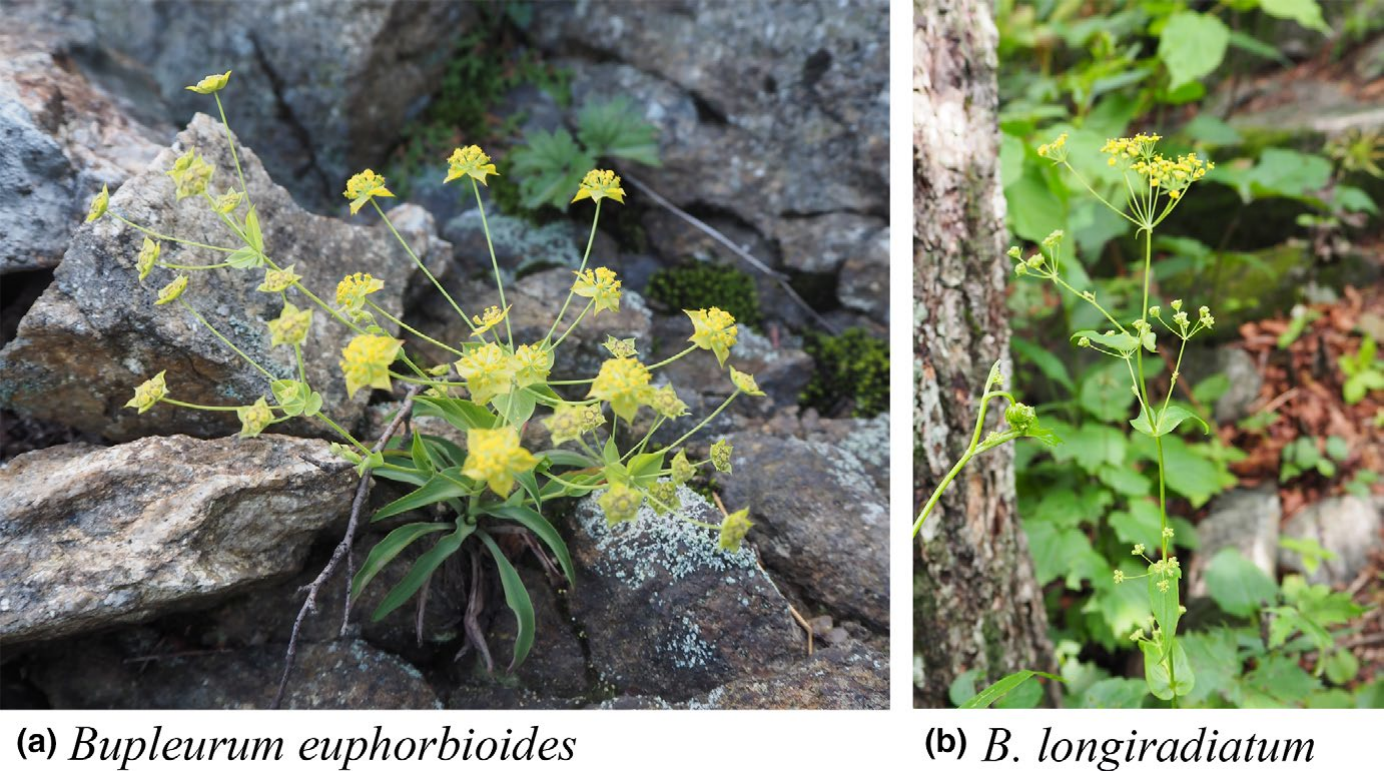
Photographs of study species taken by Dr. Soonku So on Mt. Seorak, South Korea. (a) Habitat on a rocky slope; (b) habitat under a temperate tree

**FIGURE 2:**
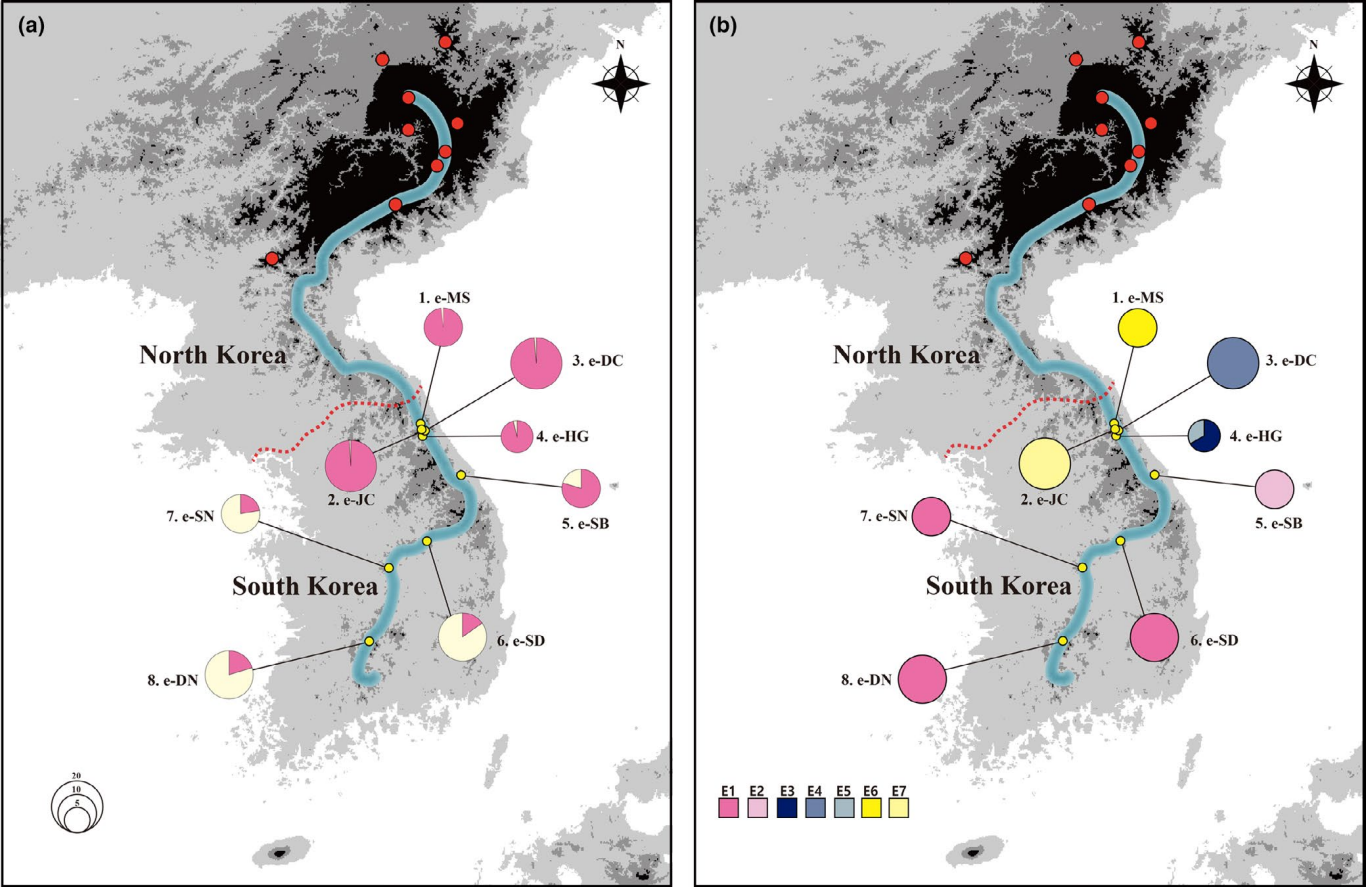
(a) Genetic composition of *Bupleurum euphorbioides* geographic populations, based on STRUCTURE clustering results using 12 microsatellite loci (*K* = 2). (b) Geographical distribution of seven chloroplast haplotypes based on three non‐coding cpDNA regions. Red points are locations where *B. euphorbioides* specimens have been collected. In addition, some populations also occur near Vladivostok (Russia). The red dotted line represents the Military Demarcation Line. The blue line indicates the Baekdudaegan mountain range

*Bupleurum euphorbioides* Nakai (Apiaceae) is a cold‐adapted annual or biennial herbal plant that is distributed on rocky slopes at the timberline (ca. 1,500 m above sea level); it is restricted to high‐elevation peaks along the main Baekdudaegan ridge on the Korean Peninsula, both in South and North Korea (Figures 1 and 2). Although the plants also occur further north than Baekdudaegan—mostly around Mt. Baekdu in northeastern China and Vladivostok in the Russian Far East (notably, it occurs at lower altitudes in these regions)—most of its range occurs in Baekdudaegan. Its habitat is severely fragmented and threatened by human disturbance, such as increasing mountain tourism in South Korea, and by overgrazing by wild animals (Kim & Chang, 2005). Therefore, *B. euphorbioide*s is internationally considered to be endangered (“EN” status) based on the IUCN Red List of Threatened Species (Kim, Kim, & Son, 2016), and it is locally managed as vulnerable (“VU” status) in North Korea (Ju et al., 2016). In contrast, *B. longiradiatum* Turcz., a congener, is widespread at mid‐elevations along the Baekdudaegan range and has a large distribution spanning from Mongolia to northeastern China, the Russian Far East, and Japan (Ohba, 1999; Sheh & Watson, 2005; Shishkin, 1950).

Plant genetic studies are often conducted by comparing congeneric species (Chung et al., 2013; Kim & Chang, 2005). This makes it possible to interpret results in an evolutionary framework. Comparisons of congeneric species allow for partial control of “background variation” caused by differences in life history traits, particularly breeding systems and seed dispersal mechanisms (Chung et al., 2012). We speculate that the distribution of *B. euphorbioides*, which might have been continuous and extensive during glacial periods, now comprises isolated fragments as a result of postglacial vertical migration; similar patterns have been observed for other cold‐tolerant plants (Gao et al., 2015; Tian et al., 2010). We therefore compared *B. euphorbioides* to *B. longiradiatum*, which has undergone range contraction during glacial periods (Zhao, Wang, Ma, Liang, & He, 2013).

In this study, we examined the possibility that the current habitats of *B. euphorbioides* are interglacial refugia, paying particular attention to the rear‐edge populations. The genetic structures were analyzed using previously developed microsatellite markers and chloroplast DNA sequences. Ecological Niche Modeling (ENM) was employed to determine the maximum contraction stage of *B. euphorbioides*’ geographic range. Furthermore, we characterized the genetic characteristics of the rear‐edge populations to assess their contribution to the species’ genetic diversity. Finally, we provide conservation guidelines for the recovery and management of threatened *B. euphorbioides* populations in the boreal and temperate climatic zones of the Baekdudaegan range.

## 2 MATERIALS AND METHODS

### 2.1 Population sampling

We collected 118 leaf samples from the eight known *B. euphorbioides* populations in South Korea, representing a significant part of its entire distribution (Figure 2). A mean of 14.5 individuals was sampled (range: 6–20) for each of eight populations. In addition to South Korea, other populations occur elsewhere, but we were unable to sample them. To reduce negative impacts on the species, the leaf samples comprised only a 2 cm long tip from each sampled leaf. The leaf tips were dried in silica gel. To avoid collecting identical clones, we collected leaves from plants more than ca. 2 m apart. Because we sampled each population extensively, our sample size is essentially the total population size. The populations on Mt. Seorak are ca. 1–8 km apart and are fragmented and isolated from one another. We regarded isolated populations between which seed dispersal could not occur as one independent populations. We were unable to find any *B. euphorbioides* individuals at a location on Mt. Odaesan where we had previously located and monitored them; that population is presumed to be locally extinct. To be able to compare patterns of genetic variation in *B. euphorbioides* and *B. longiradiatum*, we also collected 49 *B. longiradiatum* leaf samples (mean: 6.83) from the six populations in South Korea.

### 2.2 DNA extraction, genotyping, and sequencing

Total genomic DNA was extracted from the samples using MG Plant Genomic DNA Extraction SV Miniprep Kit (Macrogen Inc., Seoul, Republic of Korea) according to the manufacturer's protocol. The extracted DNA was confirmed by electrophoresis, and the concentration was measured using a Nano‐300 micro spectrophotometer, to obtain the same concentration of template DNA in each sample; this was then diluted to 15 ng of DNA for analysis.

For the microsatellite analysis, we used 12 nuclear microsatellite markers, Bul007, Bul008, Bul010, Bul015, Bul016, Bul027, Bul030, Bul032, Bul040, Bul045, Bul049, and Bul050, previously developed for *B. euphorbioides* and *B. longiradiatum* (Lee, Yoon, Han, & Kim, 2018). PCR amplification was performed as described in Lee et al. (2018). The PCR products were analyzed using an ABI 3730*xl* DNA Analyzer, with the GeneScan™ 500 LIZ™ Size Standard (Applied Biosystems). The allele sizes and peaks were determined via Peak Scanner v. 2 software (Applied Biosystems).

For our preliminary cpDNA analysis, we sequenced three chloroplast non‐coding regions from two individuals per population (a total of 16 individuals in the eight populations), using universal makers widely used in analyzing *Bupleurum* phylogeny (Ma, Zhao, Wang, Liang, & He, 2015). The preliminary analysis showed that *trn*L–*trn*F and *rpl*32–*trn*L, but not the rps16 region, were unsuitable because they contained PolyT or PolyA sequences, or had low levels of intraspecific genetic differentiation. Thus, we designed two primer pairs, after selecting regions that exhibit polymorphism within the species, using the cpDNA genomic data of *B. euphorbioides* and *B. longiradiatum*: (a) trnS‐*F* (5′‐CTTTAGTCCACTCAGCCATC‐3′) and trnG‐R (5′‐CCACTAAACTATACCCGCC‐3′); (b) ndhA‐*F* (5′‐CGCTATTACAGAACCGTACA‐3′) and ndhA‐R (5′‐CCTATGTACAAGAGTTCAGTGA‐3′). Finally, we designed and used three non‐coding chloroplast DNA regions: the *rps*16 intron (Wang et al., 2008), the *trn*S^GCU^–*trn*G^UCC^ region of the large single copy, and the *ndh*A intron of the small single copy. PCR reactions were conducted in a total volume of 20 μl using a mixture containing 15–20 ng/µl of gDNA, 2 μl of 10× *Taq* buffer with MgCl_2_, 1.6 μl of 2.5 mM dNTP, 1 μl of each primer (10 pmol), 0.25 µl of *Taq* polymerase, and sufficient distilled water. Amplification was performed as follows: initial denaturation at 95°C for 10 min; 35 cycles of denaturation at 94°C for 1 min, annealing at 52°C for 1 min, and extension at 72°C for 1 min; and a final extension at 72°C for 7 min. The PCR products were visualized on 1% agarose gels dyed with Redsafe (120 V, 25 min), purified using a Wizard SV Gel and PCR Clean‐UP system (Promega), and sequenced using an ABI 3730*xl* DNA Analyzer (Applied Biosystems). The sequences of the three regions were aligned using the Clustal algorithm (Thompson, Higgins, & Gibson, 1994). The three plastid fragments were then combined and manually adjusted using Geneious v. 10.2.3 software. Based on the concatenated sequence alignments, the cpDNA haplotypes were determined. The sequences were deposited in the GenBank database (accession numbers MT188515–MT188552).

### 2.3 Data analysis

Before analyzing the microsatellite data, two individuals identified as homozygous were randomly selected and sequenced by PCR amplification for each locus to account for the presence of null alleles and confirm the accuracy of the markers. The results of the sequencing showed that all markers perfectly amplified their target loci for both samples. We also estimated the null allele frequency using INEst (Inbreeding/Null allele Estimation) software, which calculates the null allele frequency regardless of the effect of inbreeding (Chybicki & Burczyk, 2009). This analysis showed that the null allele frequency of all the loci examined ranged from 1.5% to 6.7%. Therefore, we used all 12 microsatellite markers for statistical analysis. For *B. euphorbioides*, we classified the genetic dataset into two subsets (central populations: e‐MS, e‐JC, e‐DC, e‐HG, and e‐SB; rear‐edge populations: e‐SD, e‐SN and e‐DN), reflecting the geographical distribution based on the pronounced curvature of the Baekdudaegan range. By comparison, in *B. longiradiatum*, no geographical division was attempted, other than to compare the genetic patterns.

Population genetics summary statistics for each species were computed using GenAlEx 6.5 (Peakall & Smouse, 2006). These included the number of alleles, mean expected heterozygosity (*H*_E_), mean observed heterozygosity (*H*_O_), number of private alleles (*P*_A_), and fixation index (*F*_IS_). Allele richness (*A*_R_) and genetic differentiation among populations (*F*_ST_) were determined by calculating the overall *F*_IS_ according to the method of Weir and Cockerham (1984), using FSTAT 1.2 (Goudet, 1995). The statistical significance of *F*_ST_ was tested using the log‐likelihood (*G*)‐based exact test in FSTAT. To test for departures from Hardy–Weinberg equilibrium (HWE) and linkage equilibrium, we conducted exact tests based on a Markov Chain method (1,000 permutations), using GENEPOP 4.0 (Rousset, 2008). The possibility of recent reductions in effective population size was detected using BOTTLENECK 1.2.02 (Cornuet & Luikart, 1996) (10,000 iterations). We utilized two models for evolution—a two‐phase model (TPM) and a stepwise mutation model (SMM)—in a BOTTLENECK analysis that included the Bayesian Wilcoxon signed‐rank test, to evaluate departures from a 1:1 deficiency/excess ratio (Luikart, Allendorf, Cornuet, & Sherwin, 1998).

To infer the population structure of each species, we used a Bayesian clustering approach implemented in STRUCTURE 2.3, as calculated from microsatellite markers (Pritchard, Stephens, & Donnelly, 2000), using 1,000,000 Markov Chain Monte Carlo (MCMC) iterations (100,000 burn‐in, with admixture). The simulation used 20 iterations, with *K* = 1 to *K* = 8 clusters for *B. euphorbioides*, and *K* = 1 to 6 for *B. longiradiatum*. The optimal number of clusters, *K*, was found via the *K* method, using STRUCTURE HARVESTER (Earl & vonHoldt, 2012). CLUMPP v. 1.1.2 (Jakobsson & Rosenberg, 2007) with the Greedy algorithm was used to combine the membership coefficient matrices (*Q*‐matrices) from 1,000 iterations for *K* = 2 and *K* = 3, using random input orders.

For cpDNA analysis, intrapopulation genetic diversity levels were quantified using indices of nucleotide diversity per site (*π*) (Jukes & Cantor, 1969), number of haplotypes (*H*), and haplotype diversity (*H*d) (Nei & Tajima, 1983) using DnaSP 5.10 (Rozas, Sánchez‐DelBarrio, Messeguer, & Rozas, 2003). We determined the levels of genetic differentiation among populations of each species using *F*_ST_ (Weir & Cockerham, 1984) across three cpDNA regions, in ARLEQUIN v. 3.11 (Excoffier, Laval, & Schneider, 2005). The statistical significance of these values was assessed based on 1,000 permutations. A parsimony haplotype network was constructed using TCS v. 1.21 (Clement, Posada, & Crandall, 2000) with a 95% connection limit. In this analysis, all indels and inversions were treated as one‐point mutations. We estimated the divergence times for each haplotype of *B. euphorbioides* using BEAST 1.10.4 (Drummond & Rambaut, 2007) with GTR substitution model as selected by jModelTest 2.1.1 (Darriba, Taboada, Doallo, & Posada, 2012; Guindon & Gascuel, 2003). As outgroups, we used the L2 type sequences of *B. longiradiatum* and the chloroplast genome sequences from *Hydrocotyle verticillata* (NC_015818). There were no fossil data for *B. euphorbioides*, so we used an uncorrelated lognormal relaxed clock for clock models and coalescence with constant size based on the substitution rates of chloroplast sequence for most angiosperm species (mean: 2.0 × 10^–9^ s/s/y, standard deviation: 6.08 × 10^–10^) (Wolfe, Li, & Sharp, 1987; Zhang, Zhang, & Sanderson, 2013). The Bayesian Markov chain Monte Carlo (MCMC) was run for 10,000,000 generations with 10% burn‐in, sampling every 1,000 generations. The MCMC chains were checked in Tracer 1.7.1 (Rambaut, Drummond, Xie, Baele, & Suchard, 2018). The maximum clade credibility tree with mean heights was generated by TreeAnnotator 1.10.4 in the BEAST package after a 10% burn‐in. Finally, a tree showing the age of each branch was edited in FigTree 1.4.4 (http://tree.bio.ed.ac.uk/software/figtree/).

To test for the presence of isolation‐by‐distance (IBD), we used Mantel tests in GenAlEx 6.5 (Peakall & Smouse, 2006); this requires correlation analysis between the pairwise *F*_ST_ values for the microsatellite loci and cpDNA regions, and measurements of geographic distance among populations. Statistical significance was evaluated using 999 random permutations.

### 2.4 Ecological niche modeling

We modeled the current and historical potential distributions of *B. euphorbioides* using Maxent 3.4.1 (Merow, Smith, & Silander, 2013). Occurrence data covering entire the distribution of this species included eight sample localities from our study as well as 50 from published data (GBIF Secretariat, 2019). In total, 58 occurrence points were spatially rarefied using SDMtoolbox 2.4 (Brown, Bennett, & French, 2017) to reduce bias; thus, we used 33 points in the ENM. We obtained 19 bioclimatic variables (Online Resource 2) for the present, Last Glacial Maximum (LGM), and last interglacial (LIG), from Climatologies at High Resolution for the Earth's Land Surface Areas (CHELSA, http://chelsa‐climate.org/; Karger et al., 2017) and WorldClim v. 1.4 (http://www.worldclim.org/; Hijmans, Cameron, Parra, Jones, & Jarvis, 2005). We obtained elevation data for the present—the Global Multi‐resolution Terrain Elevation Data (GMTED2010) data set (Danielson & Gesch, 2011)—from the USGS EROS Archive (https://www.usgs.gov/land‐resources/eros/coastal‐changes‐and‐impacts/gmted2010), and for the LGM from CHELSA. To reconstruct the historical distributions, we utilized three past climate models for LGM: the Community Climate System Model (CCSM4; Gent et al., 2011), the Earth System Model based on the Model for Interdisciplinary Research On Climate (MIROC‐ESM; Watanabe et al., 2011), and The Max Planck Institute for Meteorology Earth System model (MPI‐ESM‐P). We selected one climate variable and elevation data sharing a high Spearman correlation efficient (> 0.9) using SDMtoolbox 2.4 (Brown et al., 2017) to avoid multicollinearity problems. Therefore, 11 of 20 variables were used in the ENM. To reduce the effects of uncertainty in the historical climate models, we averaged the historical distributions that were based on each of the three climate models. The climate data, for 32–47.5 N and 120–137 E (30‐arcsecond resolution), were extracted using ArcGIS 10.5 (ERSI, Redlands, CA, USA). Maxent runs were performed in batch mode with these settings: create response curves, conduct jackknife tests, use 20 replicates, cross‐validation, generate logistic output, select random seeds, and use 10,000 background points and 1,000 iterations.

## 3 RESULTS

### 3.1 Genetic statistical data

Genetic diversity parameters are shown in Table 1. For *B. euphorbioides*, we detected 107 alleles for the 12 microsatellite loci, ranging from 3 to 18 alleles per locus. In the eight *B. euphorbioides* populations, the number of alleles ranged from 21 to 67 (mean of 40.6); *H*_E_ ranged from 0.184 (southernmost, population e‐DN) to 0.557 (e‐JC) (mean of 0.401); *A*_R_ ranged from 1.537 (e‐DN) to 3.676 (e‐HG) (mean of 2.497); *P*_A_ ranged from 0 to 10 (mean of 4.375); *F*_IS_ ranged from −0.021 (e‐SN) to 0.515 (e‐DN) (mean of 0.192). The Hardy–Weinberg exact test revealed significant deviation from HWE in four of the *B. euphorbioides* populations. For *B. longiradiatum*, *H*_E_ was 0.328–0.556 and *A*_R_ was 2.167–3.280; mean *H*_E_, *A*_R_, and *F*_IS_ values were 0.474, 2.754, and 0.106, respectively.

**TABLE 1:** Genetic variation based on 12 microsatellite loci and three chloroplast non‐coding regions in 14 populations of *Bupleurum euphorbioides* and *B. longiradiatum*

Taxonomy	Pop ID	Location	Microsatellite analysis						cpDNA analysis				
			*N*_msat_ ^a^	*A*_R_	*P*_A_	*H*_O_ (*SE*)	*H*_E_ (*SE*)	*F*_IS_	*N*_cp_		*H*	*π*	*H*_d_
*B. euphorbioides*	e‐MS	Mt. Seorak (Misiryeong)	12	2.477	2	0.347 (0.069)	0.441 (0.081)	0.218		12	E6(12)	0	0
	e‐JC	Mt. Seorak (Jungcheongbong)	20	3.253	10	0.371 (0.072)	0.557 (0.073)	0.265		21	E7(21)	0	0
	e‐DC	Mt. Seorak (Daecheongbong)	20	3.093	3	0.433 (0.090)	0.515 (0.090)	0.139		20	E4(20)	0	0
	e‐HG	Mt. Seorak (Hangyeryeong)	6	3.676	6	0.528 (0.089)	0.550 (0.086)	−0.004		6	E3(4), E5(2)	0.00066	0.533
	e‐SB	Mt. Seokbyeong	10	1.720	10	0.183 (0.060)	0.233 (0.063)	0.204		10	E2(10)	0	0
	e‐SD	Mt. Sobaek	20	2.422	4	0.325 (0.048)	0.460 (0.065)	0.216		20	E1(20)	0	0
	e‐SN	Mt. Songni	10	1.796	0	0.283 (0.098)	0.266 (0.079)	−0.021		10	E1(10)	0	0
	e‐DN	Mt. Deogyu	18	1.537	0	0.097 (0.038)	0.184 (0.062)	0.515		19	E1(19)	0	0
	Mean		14.5	2.497	4.375	0.321	0.401	0.192		14.75	1.12	0.00008	0.067
*B. longiradiatum*	l‐AS	Mt. Seorak (Ansan)	6	2.797	3	0.458 (0.085)	0.512 (0.077)	0.063		8	L2(8)	0	0
	l‐OS	Mt. Seorak (Osaek)	6	3.280	3	0.458 (0.071)	0.556 (0.075)	0.128		8	L1(6), L7(2)	0.00016	0.429
	l‐OG	Mt. Odae (Guryongnyeong)	5	2.672	5	0.292 (0.086)	0.328 (0.069)	0.076		4	L6(3), L13(1)	0.00041	0.500
	l‐OD	Mt. Odae (Durobong)	4	2.167	1	0.400 (0.095)	0.442 (0.076)	0.141		4	L12(1), L14(3)	0.00021	0.500
	l‐SY	Mt. Sobaek	12	3.011	8	0.403 (0.072)	0.515 (0.075)	0.232		16	L3(4), L4(7), L5(1), L8(2), L9(2)	0.00029	0.758
	l‐DH	Mt. Deogyu	8	2.599	3	0.500 (0.088)	0.488 (0.055)	−0.002		9	L10(4), L11(5)	0.00023	0.556
	Mean		6.83	2.754	3.833	0.419	0.474	0.106		8.17	2.33	0.00022	0.457

^a^ *N*_msat_, number of individuals; *A*_R_, allelic richness; *P*_A_, number of private alleles; *H*_O_, observed heterozygosity; *H*_E_, expected heterozygosity; *F*_IS_, inbreeding coefficient. *N*_cp_, number of individuals; *H*, number of haplotypes; *π*, nucleotide diversity; *H*_d_, haplotype diversity.

The chloroplast non‐coding regions of *B. euphorbioides*, *rps*16, *trn*S^GCU^–*trn*G^UCC^, and *ndh*A, produced 19 polymorphic sites. Among these, the *trn*S‐*trn*G (727–732 bp) region was the most variable, with nine polymorphisms, a polynucleotide repeat‐length of 5 bp, an indel, and a nucleotide substitution. The *rps*16 region (854–856 bp) had five polymorphic sites (three substitutions and three indels), and the *ndh*A region (825 bp) had four polymorphic sites (all substitutions). For *B. euphorbioides*, the combined cpDNA sequences provided a total of seven haplotypes (E1–E7), aligned with a consensus length of 2,415 bp (Table S1). For *B. longiradiatum*, the combined cpDNA sequences varied in length from 2,413 to 2,469 bp and were aligned with a consensus length of 2,488 bp; in total, 14 haplotypes (L1–L14) were identified based on 24 polymorphisms (six nucleotide substitutions and 18 indels) (Table S2).

### 3.2 Genetic diversity and differentiation in *B. euphorbioides*

Relative to *B. longiradiatum*, within‐population genetic diversity was relatively lower in *B. euphorbioides*: *A*_R_ = 2.497, *H*_E_ = 0.401, *H* = 1.12, and *H*_d_ = 0.067. In *B. euphorbioides*, genetic differentiation among populations (microsatellite: *F*_ST_ = 0.372, *p* < .001; cpDNA: *F*_ST_ = 0.991, *p* < .001) was higher than in *B. longiradiatum* (microsatellite: *F*_ST_ = 0.175, *p* < .001; cpDNA: *F*_ST_ = 0.700, *p* < .001). In *B. euphorbioides*, *F*_IS_ was high, with a mean of 0.192 (Table 1).

At the regional level, for *B. euphorbioides*, the within‐population genetic variation was higher, in terms of both the microsatellite and cpDNA data, in the central populations (*A*_R_ = 2.844, *P*_A_ = 6.2, *H*_E_ = 0.459, *H* = 1.20, and *H*_d_ = 0.107), than in the rear‐edge populations (*A*_R_ = 1.918, *P*_A_ = 1.3, *H*_E_ = 0.303, *H* = 1.000, *H*_d_ = 0.000). Similarly, genetic diversity was higher for the pooled central populations (Table 2). Consistent with these findings, we detected the signatures of recent bottlenecks in the following populations: e‐SD (under TPM and mode shift models of evolution), e‐SN (mode shift), and e‐DN (mode shift); these are all rear‐edge populations (Table 3). The genetic differentiation of the central populations was lower for the microsatellite (*F*_ST_ = 0.229, *p* < .001) than cpDNA (*F*_ST_ = 0.986, *p* < .001) analysis, whereas the opposite was true for the rear‐edge populations (microsatellite: *F*_ST_ = 0.518, *p* < .001; cpDNA: *F*_ST_ = 0.000, *p* = 1).

**TABLE 2:** *Bupleurum euphorbioides* genetic diversity estimated per region, based on microsatellite loci and cpDNA haplotypes

	Microsatellite analysis				cpDNA analysis		
	*A*_R_ ^a^	*P*_A_	*H*_E_	*F*_ST_	*H*	*H*_d_	*F*_ST_
Central region							
Average/overall	2.844	6.2	0.459	0.229*	1.2	0.107	0.986*
Pooled populations	8.031	60	0.603	–	6	0.779	–
Rear‐edge							
Average/overall	1.918	1.3	0.303	0.518*	1	0.000	0.000
Pooled populations	3.917	5	0.548	–	1	0.000	–

^a^ *A*_R_, allelic richness; *H*_E_, expected heterozygosity; *P*_A_, number of private alleles; *F*_ST_, fixation index; *H*, number of haplotypes; *H*_d_, haplotype diversity.

* *p* < .001.

**TABLE 3:** Probability of a genetic bottleneck (estimated using BOTTLENECK) for eight *Bupleurum euphorbioides* populations, based on a two‐phase model (TPM) and stepwise mutation model (SMM)

Population	Wilcoxon test		Mode shift
	TPM	SMM	
e‐MS	0.61768	0.79346	No
e‐JC	0.74072	0.97876	No
e‐DC	0.75391	0.98389	No
e‐HG	0.55078	0.74023	No
e‐SB	0.81250	0.94531	No
e‐SD	0.00806*	0.06152	Yes
e‐SN	0.28906	0.53125	Yes
e‐DN	0.34375	0.50000	Yes

* Statistically significant excess heterozygosity.

### 3.3 Population genetic structure

**FIGURE 3:**
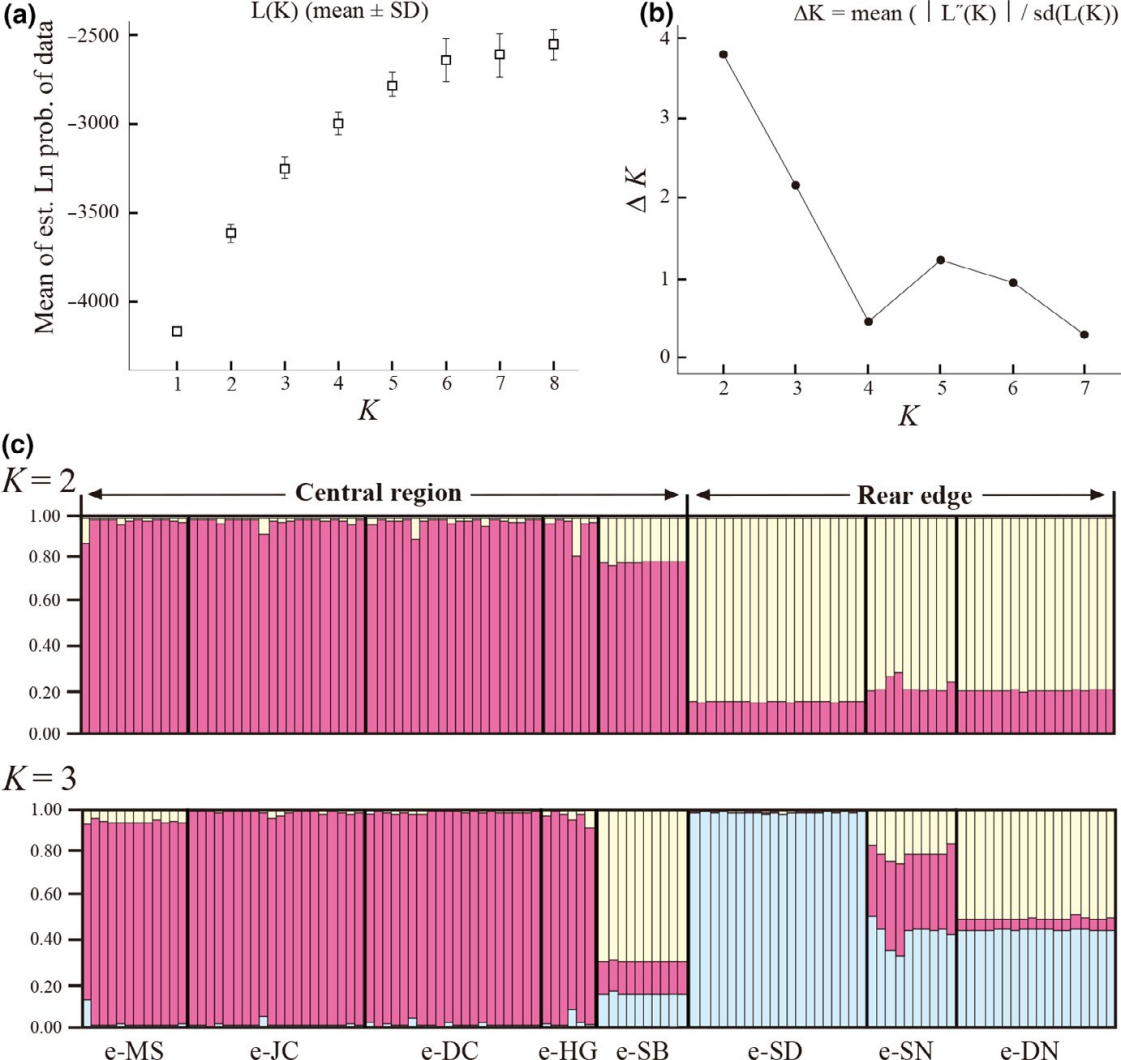
Plots showing (a) the mean log‐likelihood of the data [L(K)], (b) Evanno's delta *K* statistic, and (c) Bayesian clustering analysis of *Bupleurum euphorbioides* data conducted using STRUCTURE HARVESTER 2.3.2

**FIGURE 4:**
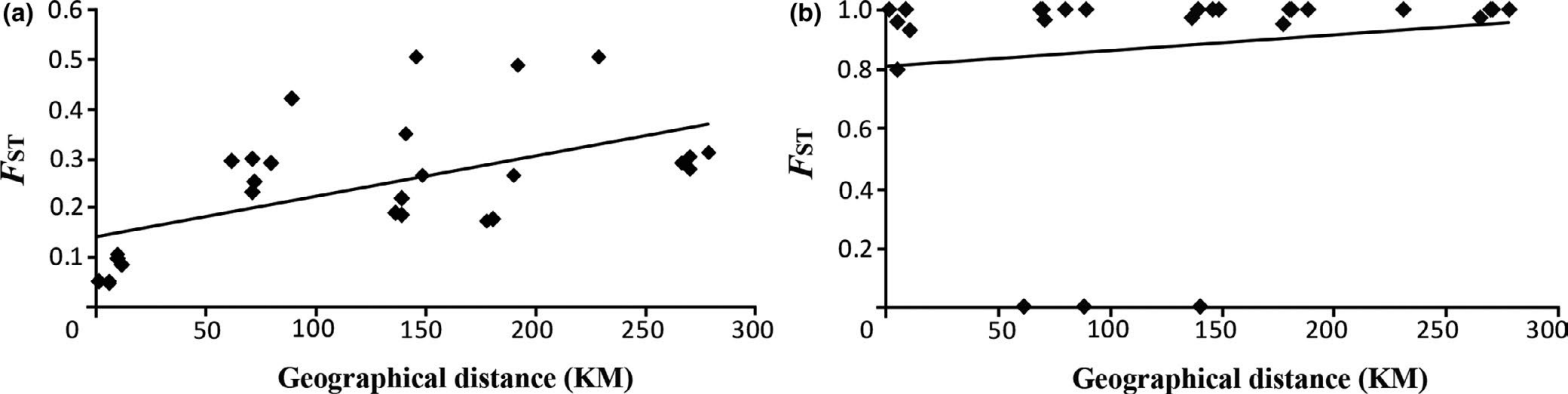
Mantel tests of the relationships among genetic differentiation (*F*_ST_ values) and geographical distance for the eight populations of *Bupleurum euphorbioides*. (a) Microsatellite data (*R* ^2^ = .317, *p* = .020). (b) cpDNA (*R* ^2^ = .023, *p* = .205)

For *B*. euphorbioides, the STRUCTURE analysis showed that the mean likelihood scores increased, with *K* values of 1–8, whereas Δ*K* decreased gradually, with *K* values of 2–7 (Figure 3). However, the standard deviation (*SD*) of L(*K*) increased considerably from *K* = 4 (Figure 3a). Therefore, we chose two as the optimal number of clusters, with three being the next most optimal (Figure 3b). At *K* = 2, the *B. euphorbioides* populations were geographically divided into two genetic clusters, comprising the central and rear‐edge populations. The central populations were mostly spatially genetically uniform. However, rear‐edge populations slightly included a gene pool that dominated the central region (pink colored; Figure 3c). Clustering using *K* = 3 produced further subdivision, with e‐SD being separated from the others, in the rear‐edge populations (Figure 3c). In this regard, we found a significant positive linear relationship between pairwise *F*_ST_ and linear geographical distance based on microsatellite data (*R* ^2^ = 0.3167, *p* = .020; Figure 4a). For *B. longiradiatum,* Bayesian clustering analysis suggested a best fit of *K* = 3 clusters; this species exhibited admixing without geographic partitions (Figure S1c, S2a), and the correlation between geographic distance and genetic distance among populations was not significant (*R* ^2^ = 0.120, *p* = .240).

**FIGURE 5:**
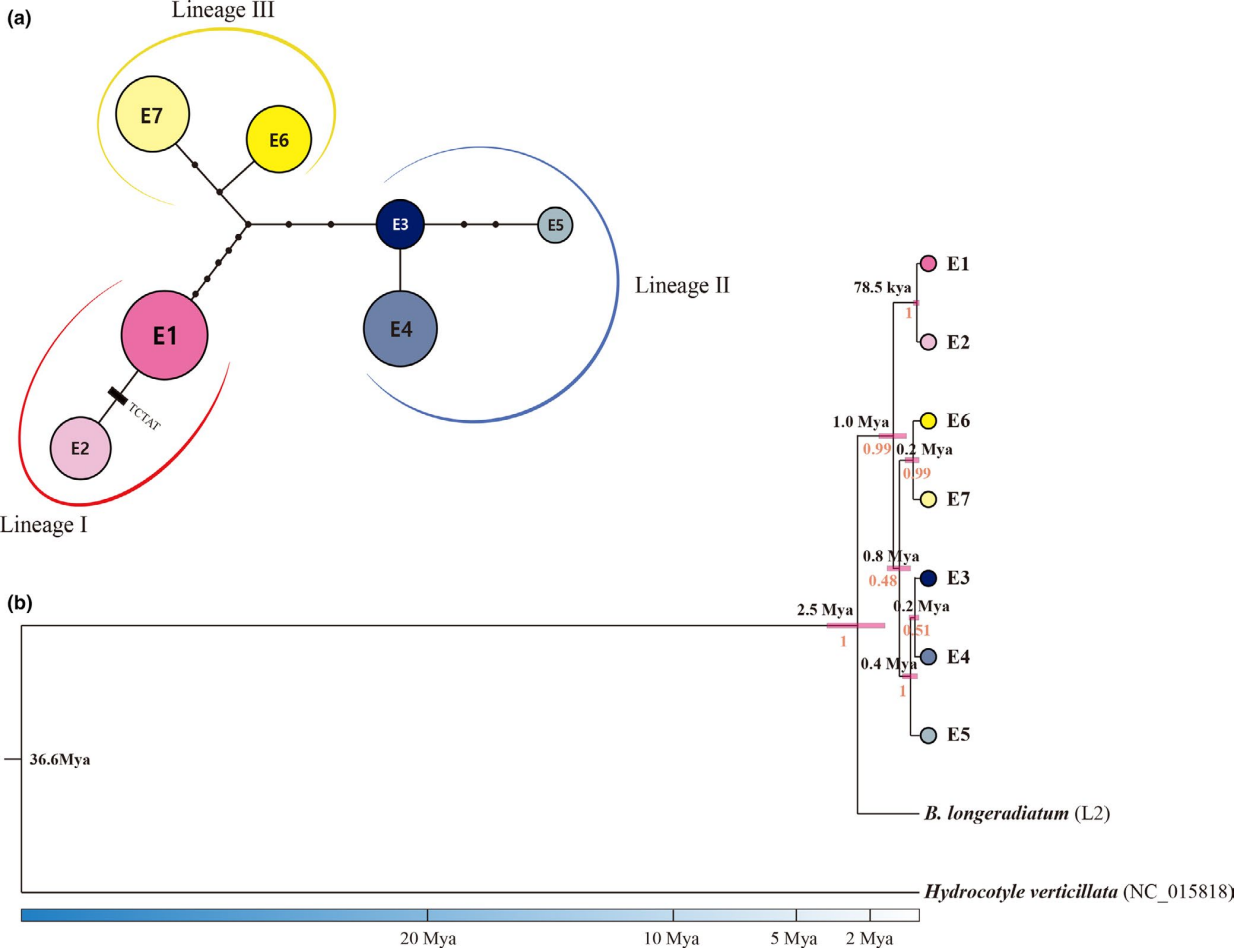
The parsimony haplotype network and Bayesian divergence time estimation based on three non‐coding regions of cpDNA. (a) The network was constructed using TCS 1.21. Small black circles are inferred intermediate haplotypes. (b) BEAST maximum credibility tree with divergence times for *Bupleurum euphorbioides*. Numbers under the branches indicate PP values and those above the line show the mean divergence time. The pink bars on nodes indicate a 95% HPD credibility interval

For *B. euphorbioides*, each population harbored only a single haplotype, except for e‐HG (Figure 2b). For *B. longiradiatum*, each population included more than two haplotypes, except for l‐AS (Figure S2). In the unrooted cpDNA parsimony haplotype network, the haplotypes showed a radial topology with three distinct lineages (Figure 5a). Lineage I, comprising haplotypes E1 and E2, occurred only in e‐SB, e‐DN, e‐SN, and e‐SD (populations 5–8), whereas lineages II and III occurred only in e‐MS, e‐JC, e‐DC, and e‐HG (populations 1–4; Figure 5b). Finally, the central and rear‐edge populations were approximately separated latitudinally, showing a spatial genetic structure. Population e‐SB (a central population) harbored only haplotype E2, derived from haplotype E1 of lineage I. From a geographical point of view, the e‐SB population showed a slight overlap between the rear‐edge and central populations in terms of the microsatellite markers and chloroplast DNA sequences examined (Figure 2). Based on the Bayesian estimates, haplotypes of *B. euphorbioides* formed a monophyletic cluster that is sister to *B. longiradiatum* (2.5 Mya) (Figure 5b). The Bayesian inference tree topology coincided with the tree from the TCS analysis. The split between lineage I and lineages II and III was dated at 1.0 Mya (0.5–1.6, 95& HPD) with high posterior probability (≈1). Although lineages II and III were separated 0.8 Mya (0.4–1.3, 95& HPD), the posterior probability was low (0.48). In contrast, the parsimony haplotype network of *B. longiradiatum* showed three distinct lineages with mixed geographic distribution (Figures S2b and S3b). The Mantel tests revealed an IBD spatial genetic structure for the two species, with no significant correlation between geographic distance and genetic distance among populations (*B. euphorbioides*: *R* ^2^ = .023, *p* = .205; *B. longiradiatum*: *R* ^2^ = .034, *p* = .350).

### 3.4 Ecological niche modeling

**FIGURE 6:**
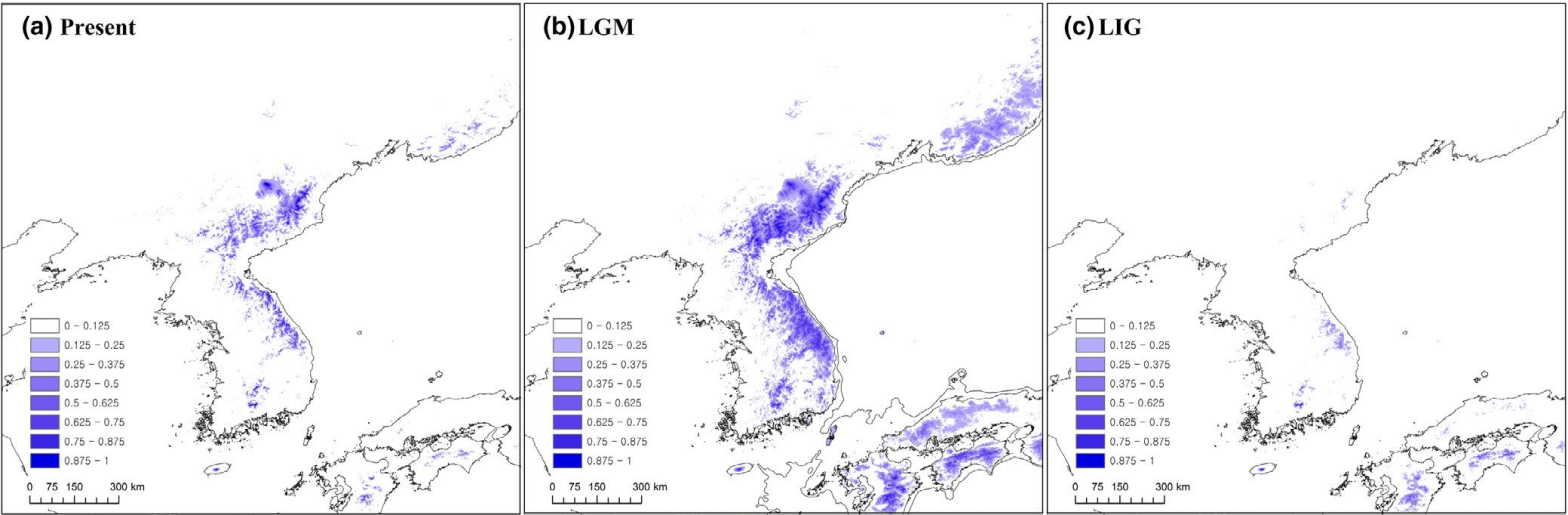
Potential distributions of *Bupleurum euphorbioides* during (a) the present, (b) the Last Glacial Maximum, LGM, and (c) the last interglacial, LIG. Distributions predicted by ecological niche modeling; potential distribution during the LGM was averaged from three general circulation models

The ENM of *B*. *euphorbioides* (Figure 6) had a high average AUC (0.967), supporting its predictive power. The most important variable was elevation (49.8% contribution), followed by bio_05 (maximum temperature of warmest month; 28.5%) and bio_04 (temperature seasonality; 7.7%). The estimated LGM distribution extended farther north‐east than did the LIG distribution, and it expanded into the lowlands via altitudinal migration in the southern part of the Baekdudaegan range (Figure 6b). The estimated distribution during the LIG, however, was narrower than the current distribution. The most likely distribution of *B*. *euphorbioides* during the LIG is almost consistent with the distribution in the Baekdudaegan mountains of the populations that we studied (Figure 6c).

## 4 DISCUSSION

### 4.1 Interglacial endurance of *Bupleurum euphorbioides*

We found that the levels of genetic variability between these congeneric species were conspicuously different. Within‐population genetic variation was low or absent in the range‐restricted *B. euphorbioides*, based on both the microsatellite and cpDNA analyses. In contrast, it was high in the widespread *B. longiradiatum*, which showed low genetic differentiation among populations. Given that these species have almost identical life histories (Sheh & Watson, 2005), these contrasting patterns of genetic diversity suggest that historical factors are the most relevant factors. Therefore, we suggest that these species have different evolutionary histories. Previous findings show that the distribution of *B. longiradiatum* was fragmented during the last glacial period into two LGM refugia, in the Qinling and Changbai Mountains in China, and expanded during the interglacial period from these refugia (Zhao et al., 2013). Although *B. longiradiatum* did not undergo extensive latitudinal range shifts, its shift was similar to those of other East Asian temperate plant species that experienced range contractions during the Quaternary glacial periods (Bao et al., 2015; Chen et al., 2012). Our results also reveal that, in South Korea, *B. longiradiatum* has relatively high genetic diversity with geographic admixing of lineages, implying that these populations were founded by postglacial immigration from multiple glacial refugia (Petit et al., 2003), presumably in China and Japan. Alternatively, the rear‐edge of Baekdudaegan may have acted as a “glacial” refugium for *B. longiradiatum*, as it has been shown to do for other temperate plants (Chung et al., 2017).

The ENM for *B. euphorbioides* revealed a maximum range contraction to only the extremely limited southern part of the Baekdudaegan mountain range during the last interglacial (Figure 6c). By comparison, its large range expansion to the northern Russian Far East (Primorsky) occurred during the LGM, with both latitudinal and altitudinal migrations. Northeastern Asia lacked the extensive ice‐caps that were ubiquitous in northern Europe and North America during the LGM (Chung et al., 2017). Conditions were therefore favorable for *B. euphorbioides* to expand northward, supporting the ENM results. These results are in contrast to other ENM studies for species in northeastern Asia, which occupied smaller ranges during the LGM than at present (*Acer mono*: Guo et al., 2014; *Eleutherococcus senticosus*: Wang, Bao, Wang, Wang, & Ge, 2016; *Quercus mongolica*: Zeng, Wang, Liao, Wang, & Zhang, 2015). Thus, unlike such cool temperate species, we hypothesize that *B. euphorbioides* retreated to its current refugia during interglacial—not glacial—periods. This scenario can reasonably explain the evolutionary history of *B. euphorbioides*.

### 4.2 Rear‐edge Baekdudaegan mountain peaks as cryptic southern refugia

The genetic distinction between the central and rear‐edge *B. euphorbioides* populations was revealed in both the microsatellite and cpDNA analyses (Figure 2). However, genetic structuring within each region seems to have progressed quite differently. There were no shared haplotypes among the central populations, but they had a uniform gene pool according to the STRUCTURE analysis, except for population e‐SB, which was distinct according to the microsatellite analysis. In contrast, the rear‐edge populations contained one fixed haplotype (E1), although the microsatellite analysis revealed slight genetic admixture. Likewise, the two analyses produced opposite patterns of genetic differentiation when comparing *B. euphorbioides* populations within each region. Such nuclear‐chloroplast DNA discordance within regions in this species might be explained by the differences in the geographic distances of pollen dispersal (fly‐pollinated; Kim & Chang, 2005) and seed dispersal (gravity‐dispersed; Bonet & Pausas, 2004; Zając, Zając, & Tokarska‐Guzik, 2009). Although flies have a limited flight distance compared to other pollinators (Kim & Chang, 2005), the fly species in these highland regions are probably capable of flying several kilometers (Inouye, Larson, Ssymank, & Kevan, 2015). Given this species’ isolation on mountain peaks, the geographic gaps present significant barriers for seeds, no matter how close the populations are. For pollen dispersal, however, the distance of ca. 5 km between the central populations may not pose a substantial barrier. Therefore, in the central region, it appears that the genetic traces of chloroplasts are well preserved, whereas the nuclear information seems to have undergone mixing as a result of current gene flow; this is reflected in the positive correlation between genetic differentiation and geographic distance (*R* ^2^ = .317, *p* = .020, Figure 4a). By comparison, rear‐edge populations appear to reflect genetic traces caused by historical colonization events well. In general, the smaller the population size, the stronger the random genetic drift is, and this promotes gene fixation. Given that bottleneck events only occurred in three populations with only a few private alleles, one plausible explanation is that the extant rear‐edge populations are relics that migrated from a single small source. Therefore, the rear‐edge populations harbor one fixed haplotype that would have been dominant in the ancestral population.

The inferred cryptic southern refugia must be verified by tracking the lineages of populations that occurred there. The populations we observed are living in an interglacial; the current climate is likely to be long lasting (ca. 50 ka; Loutre & Berger, 2000) and the future climate may be warmer (Willis & Niklas, 2004). There is therefore no guarantee that the rear‐edge populations will survive until the beginning of the next glacial. If the unique generic characteristics of the rear‐edge populations were induced by long‐term historical processes, then the rear‐edge mountain peaks would represent cryptic southern refugia. In this regard, our results provide some evolutionary clues; for instance, the rear‐edge *B. euphorbioides* survived at least one interglacial. Our estimate suggests that lineage I diverged after the mid Pleistocene. The geographical distribution of lineage I is limited to the southern latitudes, suggesting that it originated in the rear‐edge, rather than by recolonization from other regions. The strongest evidence, however, is that a tip haplotype (E2), derived from haplotype E1, the ancestral haplotype and the only one present in the rear‐edge, also occurs in the central range. In other words, the rear‐edge certainly contributed to the higher genetic diversity of the central populations. Moreover, the distribution during the LIG inferred by ENM strongly supports the possibility that the current rear‐edge populations would have survived at that time.

Taken together, the distinct genetic structuring, the occurrence of the ancestral haplotype only in the rear‐edge populations, and the other evidence we present suggest that the current habitats of the rear‐edge *B. euphorbioides* are cryptic southern refugia. Cryptic southern refugia were proposed by Stewart et al. (2010) as interglacial refugia for cold‐adapted species. However, microsatellite‐based diversity in the rear‐edge region was lower than in the central region. Moreover, the rear‐edge contained only one haplotype. These findings contradict the widely accepted hypothesis that genetic diversity decreases as latitude increases (Hewitt, 2000), presenting evidence that at least some glacial southern refugia retain high genetic diversity in pooled regional populations (as proposed by Diekmann & Serrao, 2012). Interestingly, the fixation of a single haplotype at the rear‐edge of the *B. euphorbioides* distribution resembles the genetic footprints of warm‐adapted species on scattered islands, and in northern peripheral populations founded by postglacial northward migration from putative southern glacial refugia (Lee, Lee, & Choi, 2013).

Our study is important and novel for several reasons. First, we clearly described the genetic characteristics of rear‐edge populations while inferring interglacial refugia in a phylogeographical framework. This particular case addresses fundamental aspects of species’ survival, along with the function of interglacial cryptic southern refugia, and the mechanisms whereby they maintain genetic diversity. Second, we show that the Baekdudaegan mountain system, an important refugium for many temperate plants during glacial periods, can also play this role in interglacial periods. Low genetic diversity in populations is typically expected to negatively impact species’ reproductive fitness and evolutionary potential and, ultimately, lead to extinction (Dyke, 2008; Spielman, Brook, & Frankham, 2004). However, we believe that the genetic impoverishment, and even haplotypic uniformity, of the rear‐edge populations of the cold‐adapted *B. euphorbioides* represents strategic selection that will help it to survive the upcoming favorable environment (the next glacial). In light of the fact that all central populations have private alleles, it is highly likely that the central region also represents interglacial refugia. To determine this, future studies should test leading edge samples collected around the northern end of the Baekdudaegan range.

### 4.3 Implications for conservation

From a long‐term conservation genetics perspective, it is especially important for *B. euphorbioides* that conservation efforts should not be focused on a limited set of populations or habitats. All extant habitats, which represent interglacial refugia, have the evolutionary potential to guarantee the long‐term survival of the species. The low genetic diversity of the rear‐edge populations may in fact be beneficial to the species’ survival. Given that the *B. euphorbioides* populations we sampled here represent the species’ entire South Korean distribution, it should not be difficult to conserve all of these populations in situ. Thus, first, we recommend that all known populations be protected by law to prevent further damage by loss of individuals. An appropriate measure is to control the access roads by which people can enter (e.g., Norihisa & Suzuki, 2006; Willard, Cooper, & Forbes, 2007).

Based on the genetic distinctions, we observed between the central and rear‐edge populations, a conservation strategy should be developed to maintain the unique genetic identity of each region. In terms of contemporary genetics, conservation efforts should focus on retaining the genetic uniformity of the rear‐edge rather than increasing the genetic diversity. If artificial restoration of habitats is required, it is preferable to select a single donor population after considering its lineage. Ex situ conservation efforts may be effective in the central region (especially on Mt. Seorak) where the haplotypes are not shared among populations. Finally, the fact that pollen transfer could enable gene flow over several kilometers should be considered in restoration attempts.

## CONFLICTS OF INTEREST

The authors have no conflict of interest to declare.

## AUTHOR CONTRIBUTION

**Won‐Bum Cho:** Conceptualization (supporting); Formal analysis (lead); Methodology (lead); Resources (equal); Software (lead); Visualization (lead); Writing‐original draft (equal). **Soonku So:** Investigation (equal); Resources (equal); Validation (supporting); Visualization (supporting); Writing‐original draft (supporting). **Eun‐Kyeong Han:** Formal analysis (supporting); Methodology (supporting); Software (supporting); Visualization (supporting); Writing‐review & editing (supporting). **Hyeon‐Ho Myeong:** Investigation (equal); Methodology (supporting); Resources (equal). **Jong‐Soo Park:** Methodology (supporting); Software (equal); Visualization (supporting); Writing‐original draft (supporting). **Seung‐Hyun Hwang:** Formal analysis (supporting); Investigation (equal); Resources (supporting). **Joo‐Hwan Kim:** Conceptualization (supporting); Supervision (supporting); Writing‐review & editing (supporting). **Jung‐Hyun Lee:** Formal analysis (supporting); Methodology (supporting); Project administration (equal); Supervision (lead); Visualization (supporting); Writing‐original draft (equal); Writing‐review & editing (equal).

## Supporting information

Supplementary Material

## Data Availability

Data are available at the Dryad Digital Repository, https://doi.org/10.5061/dryad.pzgmsbchx. Additional tables and figures are presented as Supplemental Information. Sequence data are available on GenBank (accession numbers MT188515–MT188552).
