# Supplementary material for: Rear‐edge, low‐diversity, and haplotypic uniformity in cold‐adapted Bupleurum euphorbioides interglacial refugia populations
Source: Ecol Evol. 2020 Aug 24;10(19):10449–62. doi: 10.1002/ece3.6700 (PMC7548181; doi:10.1002/ece3.6700)
Supplement: Supplementary file 1 — Supplementary Material [file ECE3-10-10449-s001.docx]

**Supplemental Information**

Table S1. Variable sites of the aligned sequences of three cpDNA fragments in seven *Bupleurum euphorbioides* haplotypes (E1–E7). The Bolded number indicates large indel.

|  | Region | | | | | | | | | | | | | | | | | | | | | | | |
| --- | --- | --- | --- | --- | --- | --- | --- | --- | --- | --- | --- | --- | --- | --- | --- | --- | --- | --- | --- | --- | --- | --- | --- | --- |
|  | *rps*16 | | | | | |  | *trn*S-*trn*G | | | | | | | | | |  | | *ndh*A | | | | |
| Haplotype | 139 | 159 | 263 | 549 | 760 | 785 |  | 913 | 933 | 1048 | 1445 | 1462 | 1472 | 1487 | 1545 | 1579 |  | | 1878 | | 2332 | 2335 | 2357 |  |
| E1 | C | - | - | T | G | G |  | A | - | C | A | - | G | A | C | T |  | | C | | T | A | A |  |
| E2 | . | . | . | . | . | . |  | . | **1** | . | . | . | . | . | . | . |  | | . | | . | . | . |  |
| E3 | A | . | T | G | . | A |  | . | . | T | C | T | . | . | . | . |  | | . | | A | T | G |  |
| E4 | A | . | T | G | . | A |  | T | . | T | C | T | . | . | . | . |  | | . | | A | T | G |  |
| E5 | A | . | T | G | . | A |  | . | . | T | C | T | T | C | T | . |  | | . | | A | T | G |  |
| E6 | . | . | . | G | - | A |  | . | . | . | C | . | . | . | . | . |  | | T | | A | T | G |  |
| E7 | . | T | . | G | . | A |  | . | . | . | C | . | . | . | . | A |  | | T | | A | T | G |  |

Table S2. Variable sites of the aligned sequences of three cpDNA fragments in 14 *Bupleurum longiradiatum* haplotypes (L1–L14)*.* The Bolded numbers indicates large indel.

|  | Region | | | | | | | | | | | | | | | | | | | | | | | | | |
| --- | --- | --- | --- | --- | --- | --- | --- | --- | --- | --- | --- | --- | --- | --- | --- | --- | --- | --- | --- | --- | --- | --- | --- | --- | --- | --- |
|  | *rps*16 | | | | |  | *trn*S-*trn*G | | | | | | | | | | | | | | |  | *ndh*A | | | |
| Haplotype | 1  25 | 256 | 286 | 688 | 753 |  | 890 | 991 | 992 | 993 | 994 | 1037 | 1040 | 1041 | 1042 | 1192 | 1425 | 1449 | 1514 | 1515 | 1608 |  | 2000 | 2215 | 2350 | 2383 |
| L1 | - | - | T | T | G |  | A | - | - | - | A | C | C | - | T | T | - | - | T | T | G |  | A | **3** | - | - |
| L2 | . | T | . | . | . |  | . | . | . | A | . | . | . | . | . | . | . | . | . | . | . |  | C | . | . | . |
| L3 | . | T | . | . | . |  | . | . | . | . | . | . | . | T | . | . | . | . | . | . | A |  | . | . | . | . |
| L4 | . | T | . | . | . |  | . | . | . | . | - | . | . | T | . | . | . | . | . | . | A |  | . | . | . | . |
| L5 | . | T | . | . | . |  | . | . | . | . | . | . | . | . | . | . | . | . | . | . | A |  | . | . | . | . |
| L6 | . | T | . | . | . |  | . | . | . | . | . | . | . | . | . | C | . | . | . | . | A |  | . | . | **4** | . |
| L7 | . | T | . | G | . |  | . | . | . | . | . | . | . | . | . | . | . | . | . | . | . |  | . | . | . | . |
| L8 | . | . | . | G | . |  | . | . | . | . | . | . | - | . | - | . | . | . | . | . | A |  | . | - | . | . |
| L9 | . | T | . | . | . |  | . | A | A | A | . | - | . | . | . | . | **1** | **2** | . | . | A |  | . | - | . | 5 |
| L10 | A | . | - | . | . |  | . | . | . | . | . | - | . | . | . | . | . | **2** | - | . | . |  | . | - | . | 5 |
| L11 | A | . | - | . | . |  | . | . | . | . | . | - | . | . | . | . | . | **2** | - | . | A |  | . | - | . | 5 |
| L12 | A | T | - | . | . |  | . | . | . | . | . | . | . | . | . | . | . | . | - | - | . |  | . | - | . | 5 |
| L13 | A | T | - | . | . |  | . | . | . | . | . | . | . | . | . | . | . | . | . | . | A |  | . | - | . | 5 |
| L14 | A | T | - | . | A |  | C | . | . | . | . | . | . | . | . | . | . | . | - | - | . |  | . | - | . | 5 |

**1**: TTTTTTATTTAGTTATTTTTTTTA; **2**: TTTTTTATTTAGTTATTTT; **3**: TTTTATGCAG; **4**: TACATG; **5**: TAAAATCC


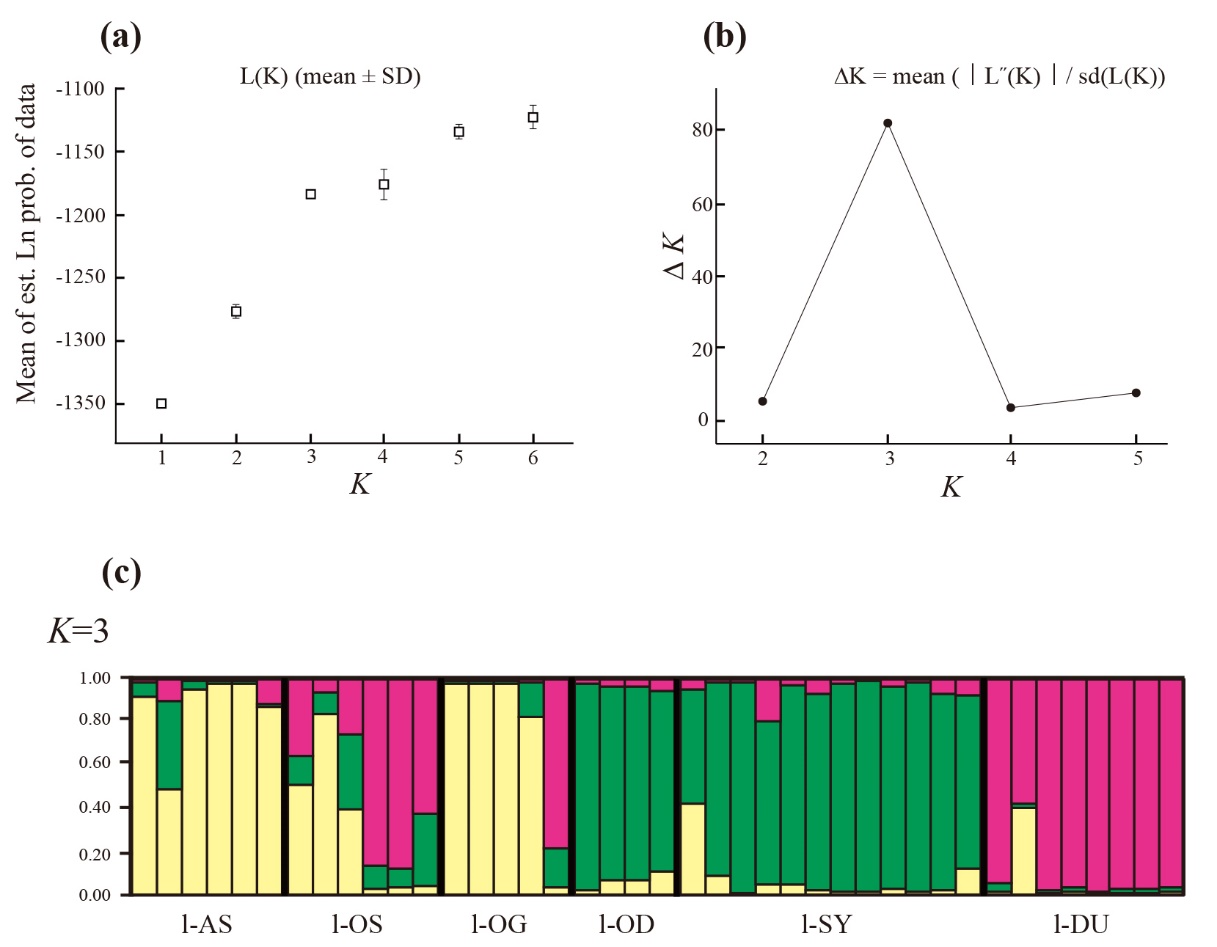


Figure S1. Plots showing (a) the mean log likelihood of the data [L(*K*)], (b) Evanno’s delta *K* statistic, and (c) Bayesian clustering analysis of *Bupleurum longiradiatum* data, conducted using STRUCTURE HARVESTER 2.3.2*.*


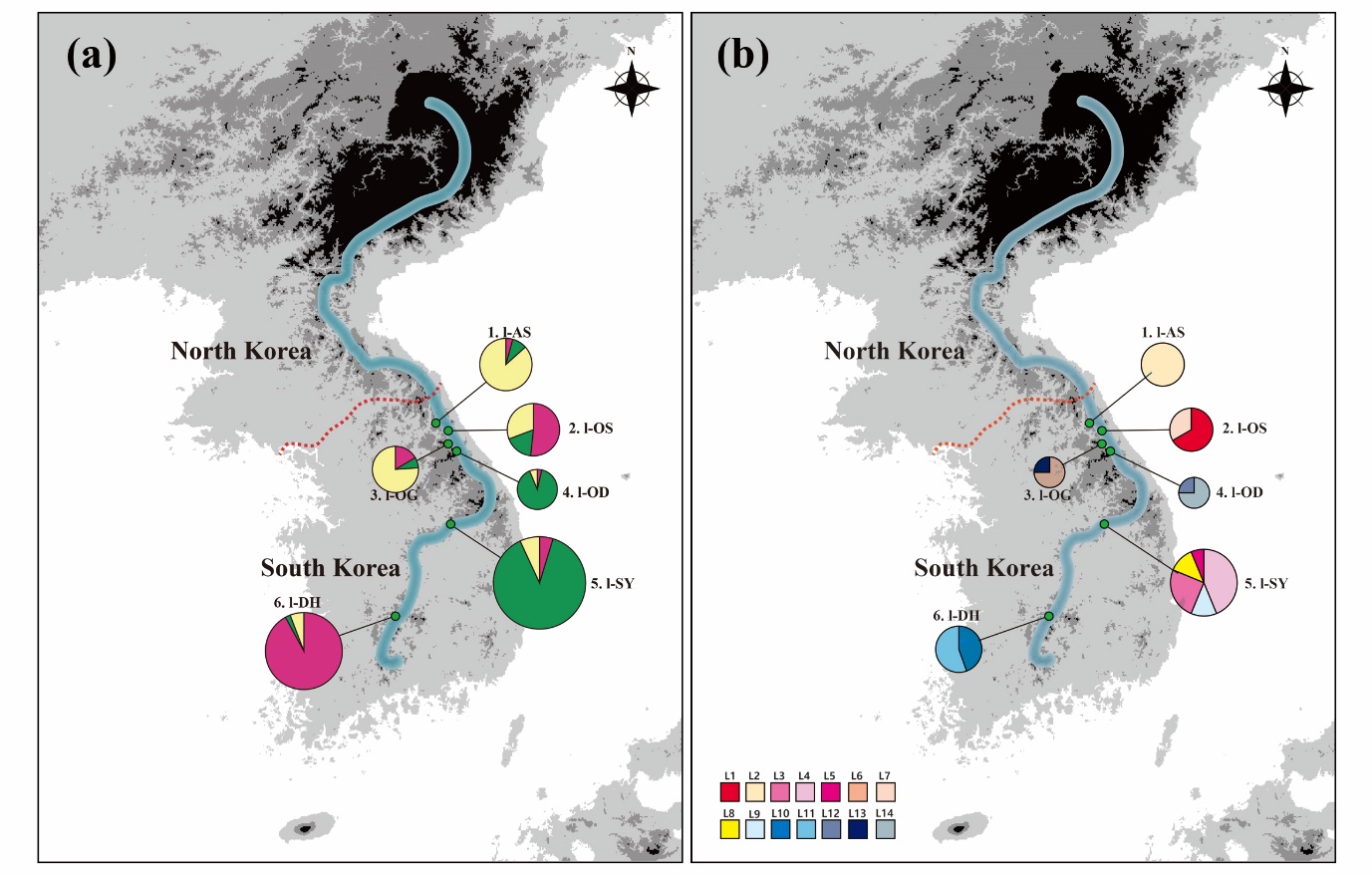


Figure S2. (a) Genetic composition of *B. longiradiatum* populations, based on STRUCTURE clustering results using microsatellite loci (*K* = 3). (b) Geographical distribution of chloroplast haplotypes. The red dotted line represents the Military Demarcation Line. The blue line marks the Baekdudaegan mountain range.


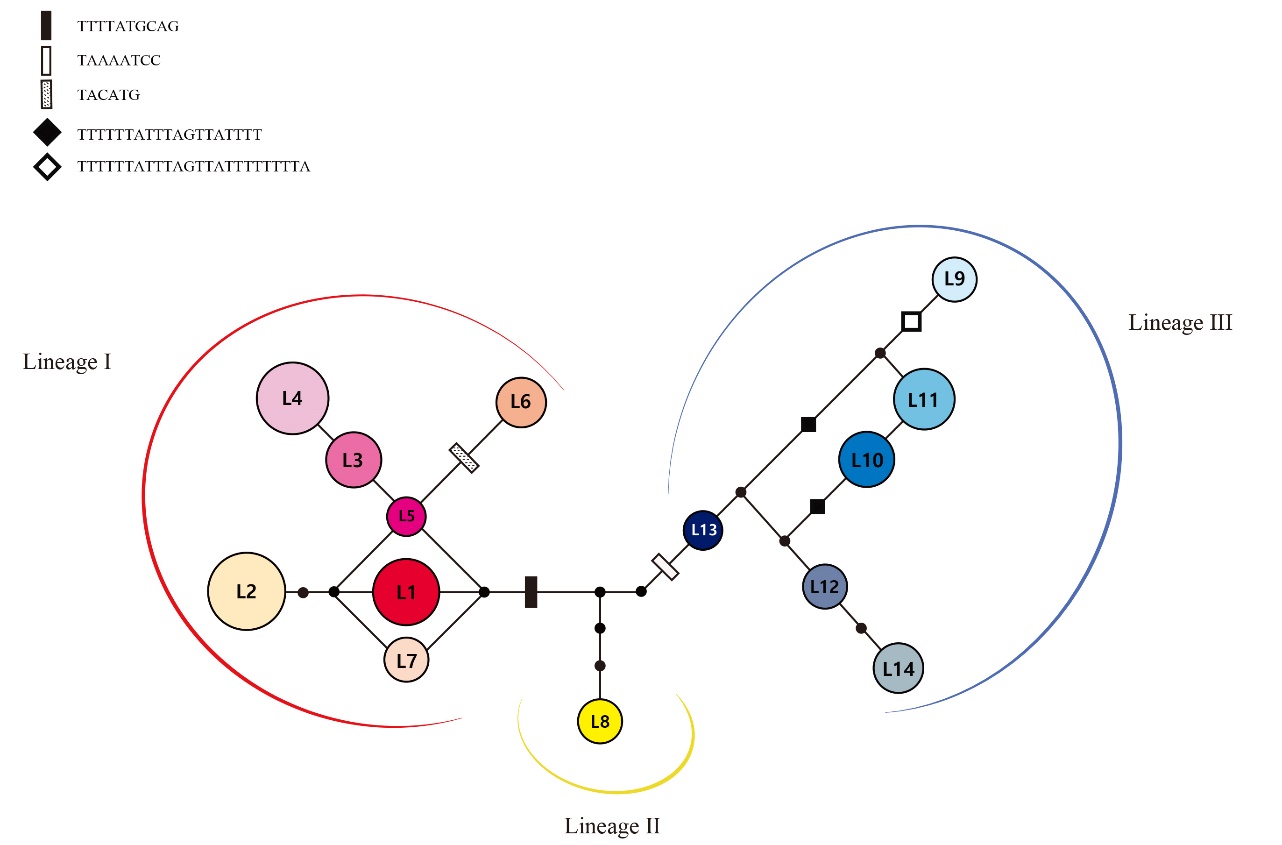


Figure S3. Parsimony haplotype network created using TCS software based on three non-coding regions of *Bupleurum longiradiatum* cpDNA. Small black circles are inferred intermediate haplotypes.
